# Effect of Ginkgolide in Ischemic Stroke patients with large Artery Atherosclerosis: Results from a randomized trial

**DOI:** 10.1111/cns.13742

**Published:** 2021-10-22

**Authors:** Yi Dong, Jingyu Zhang, Yanxia Wang, Lihong Zhao, Runhui Li, Chunhua Wei, Qingke Bai, Lishu Wan, Liping Sun, Shejun Feng, Mingyao You, Chun Wang, Hongtian Zhang, Qing He, Ming Yu, Qiang Dong, GISAA committee, Lei Huang, Wenjie Cao, Dongjuan Xu, Jianjun Guo, Jiadong Zhang, Guofang Chen, Yan Wei, Shuangxing Hou, Xijing Mao, Rongxia Ji, Jifa Long, Caixiao Chen, Yong Zhao, Guozhong Li, Wenjun Yu, Jianhua Xu, Xiangyu Pu, Xiaohong Li, Zhilin Jiang, Yi Yang, Wei Li, Qingyou Zeng, Tao Sun, Yingmin Song, Baoshen Wang, Guiru Zhang, Huisheng Chen, Junyan Liu, Zhenguo Liu, Yongge Hou, Biyun Zong, Jun Liu, Bihua Wu, Xin Wang, Ding Qin, Ming Zhang, Weizhong Gu, Houqin Chen, Qing He, Anding Xu, Yun Xu, Mingzong Yan, Xiaoya Feng, Jun Tan, Ping Sun, Zhengqi Lu, Xiao Bo, Zhen Jiao

**Affiliations:** ^1^ Department of Neurology Huashan Hospital affiliated to Fudan University Shanghai China; ^2^ Department of Neurology The Fourth Hospital affiliated to Harbin Medical University Harbin China; ^3^ Department of Neurology Hejian People’s Hospital Hejian China; ^4^ Department of Neurology Dandong People’s Hospital Dandong China; ^5^ Department of Neurology Central Hospital affiliated to Shenyang Medical College Shenyang China; ^6^ Department of Neurology Nanshi Hospital affiliated to Henan University Henan China; ^7^ Department of Neurology Pudong New Area People’s Hospital Shanghai China; ^8^ Department of Neurology Dandong First Hospital Dandong China; ^9^ Department of Neurology Pingdingshan Municipal Second People’s Hospital Pingdingshan China; ^10^ Department of Neurology Handan Central Hospital Hebei China; ^11^ Department of Neurology Guizhou Medical University affiliating Hospital China; ^12^ Department of Neurology The First People’s Hospital Ruzhou China; ^13^ Department of Neurology Zhecheng People’s Hospital Zhecheng China; ^14^ Department of Neurology Transportation Center Hospital Yunnan China; ^15^ Department of Neurology Jiangsu University affiliating Hospital Jiangsu China

**Keywords:** acute ischemic stroke, ginkgolide, intracranial stenosis, PAF

## Abstract

**Background:**

Dual antiplatelet therapy is considered beneficial in acute ischemic stroke (AIS) patients with intracranial artery stenosis (ICAS), with more bleeding events. Ginkgolide is shown to reduce platelet activation after infarction, which might be of benefit in AIS. We aimed to explore the effect of Ginkgolide in AIS patients with ICAS.

**Methods:**

This was a randomized, double‐blinded, placebo‐controlled trial conducted at 61 centers in China. Within 72 h after onset, consecutive patients diagnosed as AIS with ICAS were randomized to either Ginkgolide or placebo treatment. The primary outcome was the composite of mortality and recurrent stroke (ischemic or hemorrhagic) during first 4 weeks in an intention‐to‐treat analysis. Secondary functional outcome was assessed by modified Rankin Scale and improvement of stroke severity was assessed by National Institution of Health Stroke Scale at day 28. Safety outcome was measured by the rate of severe adverse event (SAE).

**Results:**

There were 936 patients randomized to either Ginkgolide or placebo treatment. Their average age was 64.2 ± 10.4 years old and 36.0% of the patients were female. The composite index event occurred in six patients in placebo group, and none occurred in Ginkgolide group (risk ratio 1.01; 95% CI 1.00–1.02). There were more patients who achieved favorable outcome in Ginkgolide group, compared with that of the placebo group (OR 2.16, 95%CI 1.37–3.41). SAE occurred in five (1.1%) patients in the Ginkgolide group and three (0.6%) in the placebo group (OR0.60, 95CI% 0.14–2.53). Intracranial hemorrhage occurred in 1/473 (0.2%) in the placebo group.

**Conclusions:**

Ginkgolide, working as PAF antagonist, may reduce recurrent stroke in AIS with ICAS patients within 72 hours after onset. It might be an optional treatment in moderate‐to‐severe AIS patients with ICAS. (http://www.chictr.org.cn Number as ChiCTR‐IPR‐17012310).

## Graphical abstract

Although dual antiplatelet therapy is considered benefit in acute ischemic stroke (AIS) patients with intracranial artery stenosis (ICAS), there are more bleeding events. Ginkgolide, works as PAF antagonist, may reduce recurrent and mortality in AIS with large artery stenosis within 72 hours atfer onset during 90 days follow‐up. It might play a role of neuroprotection in AIS patients with ICAS

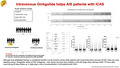

## 1 INTRODUCTION

The aggressive medical management, including dual antiplatelets treatment, to prevent stroke in non‐disabling stroke with intracranial stenosis (ICAS), has become the standard therapy since 2011.^1^, ^2^ The clopidogrel plus aspirin versus aspirin alone for reducing embolization in patients with acute symptomatic cerebral or carotid artery stenosis study (CLAIR) and Clopidogrel in High‐Risk Patients with Acute Nondisabling Cerebrovascular Events (CHANCE) trial showed the benefit of dual antiplatelet therapy (DAPT) in reducing the rates of recurrence stroke and mortality within 28–90 days.^3^, ^4^ Additionally, CHANCE subgroup analysis showed the extra benefit of DAPT in patients with ICAS.^4^, ^5^

However, the use of dual antiplatelets treatment is limited due to its timing and its higher risk of bleeding.^6^ The DAPT used could be one of the predictors to bleeding risk.^6^ In platelet‐oriented inhibition in new TIA and minor ischemic stroke (POINT) trial, the risk of hemorrhagic stroke or transformation increased if DAPT was used for 90 days.^7^ Limited evidence showed that DAPT in non‐minor stroke was not superior to monotherapy in ICAS patients during the acute phase.^8^

Ginkgolide is a promising agent that enhances antiplatelet agent function via platelet aggregation factor (PAF) inhibition.^9^, ^10^, ^11^ On the other hand, it might also have less bleeding risk. Therefore, we conducted this pilot study to compare the feasibility and efficacy of Ginkgolide in AIS patients with ICAS. The mechanism of Ginkgolide as an antagonist of PAF was also explored. Since common platelet aggregation is synergistically mediated by PAF, thromboxane A2 (TXA2), and adenosine‐5’‐diphosphate (ADP), all parameters were assessed in this study.

## 2 METHODS

### 2.1 Study Protocol and study design

The GISAA study protocol and data collection were approved by the ethics committee of Huashan Hospital (Ethical approval number: KY2016‐199) and all of the study centers.^12^ This study was conducted in accordance with the Declaration of Helsinki. The trial was a prospective, multicenter, randomized, open‐label, active controlled, blinded endpoint trial (registered as http://www.chictr.org.cn; number as ChiCTR‐IPR‐17012310). All participants or their representatives provided written consent before randomization.

### 2.2 Participates

From October 2016 to July 2018, patients with an acute moderate‐to‐severe ischemic stroke were consecutively screened at 61 centers in China. Patients aged above 18 years, with National Institutes of Health Stroke Scale (NIHSS) scored >3 points at randomization, were included within 72 h of symptom onset. All patients had a baseline MRA or CTA scan to confirm their large vessel atherosclerotic changes. The ICAS in our study was defined as a stenosis or occlusion of one or more than one of these vessels: intracranial carotid artery, anterior cerebral artery, middle cerebral artery, and posterior cerebral artery or vertebrobasilar artery stenosis or occlusion on CT/MR angiogram.

Patients were excluded from the trial if they were candidates of thrombolysis and thrombectomy, or had a diagnosis of intracranial hemorrhage, acute coronary syndrome, rheumatic heart disease, or other etiologies of stroke, such as cardioembolism, reversible cerebral vasoconstriction syndromes, vasculitis, dissection, brain tumor, parasitic disease, and other neurological disease that account for the neurological symptoms; patients with a modified Rankin Scale (mRS) score of more than 1 at randomization; or any contraindication to ginkgo, alcohol, and glycerin were excluded.^12^

### 2.3 Randomization and procedures

Immediately after signing the written informed consent, eligible patients were randomized at a ratio of 1:1 to receive either Ginkgolide or placebo. Randomized block was used to allocate patients, and the random allocation scheme of 1:1 for the treatment group and the control group was made using SAS 9.4 software. The generate random, encoding by using randomized method, block selection and the length of the random initial value parameters such as seeds of confidential data, are sealed together in blind bottom. Patients were also stratified by their baseline severity and risk factors. According to this random number, the drug is coded by the person who has nothing to do with the test. Each clinical research center was allocated according to the assigned drug number and selected according to the case order.

The trial included four visits: randomization (baseline), day 7 (±1 day), day 14 (±3 days), and day 28 (±3 day). All visits involved a face‐to‐face interview. Data were collected in a case report form.

The blood sample was drawn at baseline, day 7, and day 14 for the purpose of monitoring the dynamic changes in common platelet‐activating pathways. Levels of PAF, TXA_2_, and ADP were analyzed by the core laboratory using ELISA Kit with standard protocol. Both the investigators and the patients were blinded to the platelet reactivity data until the end of the trial.

### 2.4 Intervention

All patients who met the inclusion criteria were randomly assigned to receive aspirin 100 mg oral daily combined with intravenous ginkgolide 10 ml for 14 days or aspirin 100 mg oral daily combined with placebo after randomization immediately. Placebo was produced specifically as unidentical in appearance, and labeled with same drug label. Blinded details were demonstrated in a study protocol published previously.^12^

### 2.5 Outcome

Primary outcome was to measure the rate of stroke recurrence and mortality at day 28 after randomization. Recurrent stroke was defined as an index event (ischemic or hemorrhagic) after 24 h of randomization or NIHSS increasing >4 points. Ischemic stroke was defined as an acute focal infarction of the brain or retina with clinical or imaging evidence of infarction lasting 24 h or more, or rapid worsening of an existing focal neurological deficits with NIHSS score increasing >4 points. Hemorrhagic stroke was defined as acute‐onset brain parenchyma or subarachnoid hemorrhage with associated neurologic symptoms.

Secondary outcome included the following: (1) the improvement of clinical symptoms measured by NIHSS score at 14 and 28 days after randomization; and (2) the functional outcome assessed by mRS at 28 days after randomization.

The primary safety outcome included any severe adverse events (SAE), including major bleeding events, which were defined as that in the PLATO study: fatal or life‐threatening hemorrhage, major hemorrhage, and other.

Final imaging analysis and interpretation were performed centrally. All CTAs or MRAs were required from the individual centers in digital format and were read centrally by two readers (WJ C and LH) who were blinded to patients’ baseline characteristics, intervention, and outcome information. The presence of moderate‐to‐severe ICAS was defined as 50%–99% stenosis (Warfarin‐Aspirin Symptomatic Intracranial Disease trial criteria^13^) or occlusion of at least one of the following arterial segments on maximum intensity projections of 3D time‐of‐flight MRA: intracranial portion of internal carotid arteries, middle cerebral arteries (M1/M2), intracranial portion of vertebral arteries, and basilar artery. Disagreement in the degree of stenosis was resolved by consulting with a third neuroradiologist. The intra‐ and interrater reliabilities of detecting ICAS on MRA images at the reading center have been reported previously, which were 0.793 and 0.815, respectively.^5^

### 2.6 Sample size and Statistical analysis

The software PASS11.0 was used to calculate the sample size. The validity test was used with superiority cutoff = 0.010, α = 0.025 (single side), and β = 0.20. In addition, the calculation showed that the sample size in each group was 366 cases. Considering the possible subject dropout during clinical trial, the estimated value derived from the formula was increased by 20%; thus, the total sample size was 878 cases, 439 in each group.

The statistical analyses were performed by the Department of Epidemiology and Health Statistics, Public Health of HuaXi Institution, Sichuan University. Proportions were presented for categorical variables, and medians with interquartile ranges or means (standard deviation) were presented for continuous variables. We compared the index events at the day 28 follow‐up (the primary outcome) between the two groups using Cox proportional hazards regression. A specified secondary analysis of the categorical shift in mRS would be undertaken on the full range (0–6) of the mRS using Cochran–Mantel–Haenszel shift test and proportional odds logistic regression subject to the validity of shift analysis model assumptions. The proportion of major bleeding events (the primary safety outcome) was compared between the two study groups using logistic regression models. The data of the level of PAF, TXA2, and ADP were tested for normality. If those continuous data did not show normal/Gaussian distribution, it would be analyzed via Wilcoxon test.

### 2.7 Data availability

All individual data were de‐identified from the GISAA trial. These data would be shared with qualified researchers upon request to the principal investigator (Dr. Qiang Dong).

## 3 RESULTS

**FIGURE 1:**
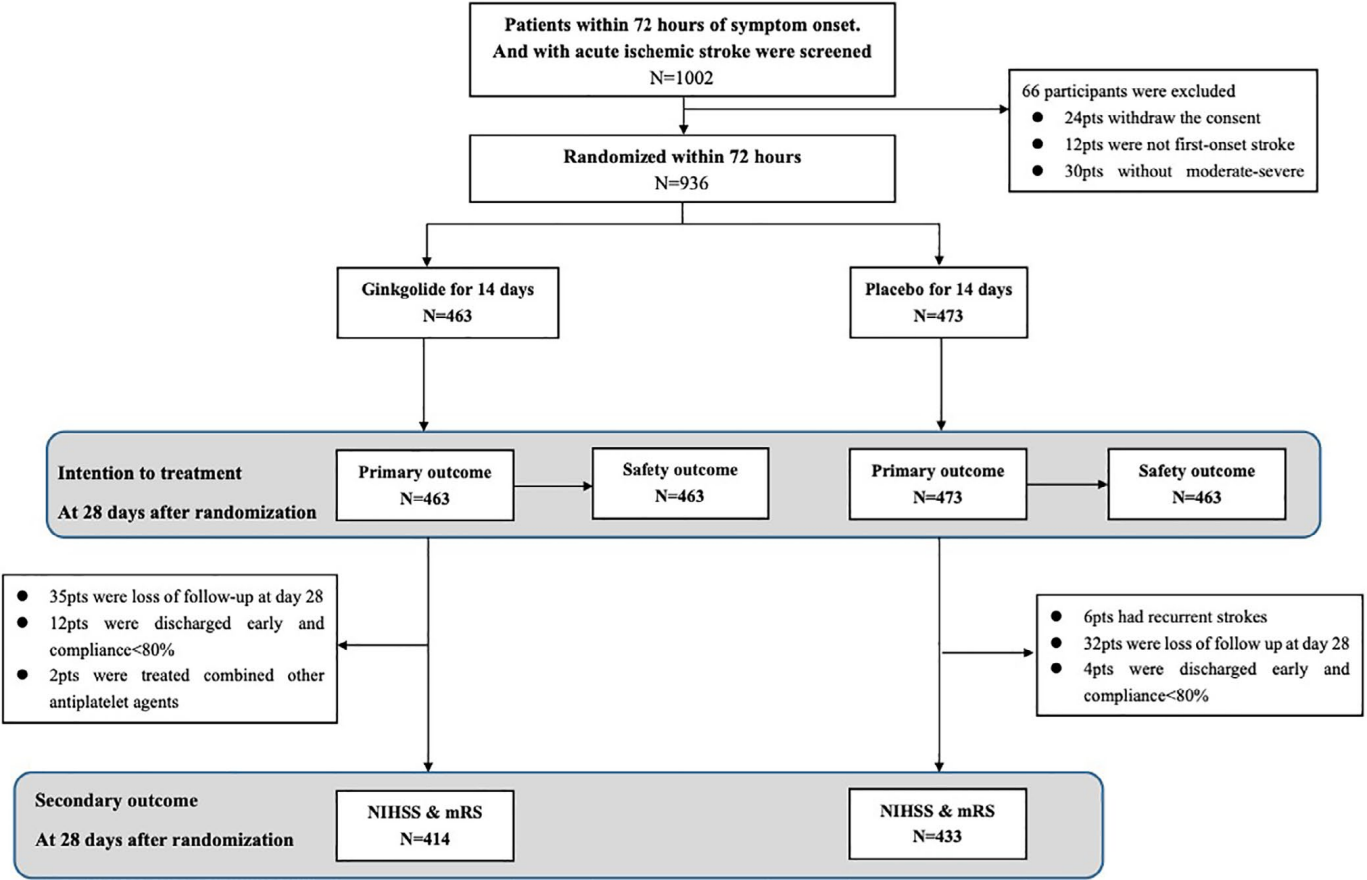
Trial profile. mRS, modified Rankin Scale; NIHSS, National Institution of Health Stroke Scale

From October 2016 to July 2018, a total of 1002 patients with an acute moderate‐to‐severe ischemic stroke were consecutively screened at 61 centers in China. Our study enrolled 936 stroke patients with ICAS within 72 h of symptom onset (the flowchart is shown in Figure 1). The average age was 64.2 ± 10.4 years (ranged from 30 to 90 years), and 36.0% of the patients were females. Their average onset to randomization time was 1.75 ± 0.83 days and the median severity of stroke was five points [interquartile as 4–7]. There were 463 patients in the ginkgolide group and 473 patients in the placebo group. The baseline characteristics of the 936 patients are shown in Table 1. The majority participants met the criteria of moderate‐to‐severe ICAS according to the central imaging center assessment. The proportion of disagreement between two neuroradiologists was 13% among all 936 patients. Then, those disagreements in the degree of stenosis were resolved by consulting with a third neuroradiologist, and we achieved the final judgment.

**TABLE 1:** The baseline characteristics at randomization

Variable	GISAA (*n* = 463)	Control (*n* = 473)
Age, years old	64.31 (10.68), 65 [57–71]	64.12 (10.40), 65 [58–71]
≥70	144 (31.10%)	142 (30.02%)
Sex, male, *n* (%)	283 (61.12%)	316 (66.81%)
Ethnic origin		
Han	459 (99.14%)	469 (99.15%)
Other	4 (0.86%)	4 (0.85%)
Time to randomization, days	1.72 (0.83), 2 [1–2]	1.77 (0.83), 2 [1–2]
(0,1)	185 (39.96%)	172 (36.36%)
(1,2)	195 (42.12%)	207 (43.76%)
(2,3)	83 (17.93%)	94 (19.87%)
NIHSS at randomization	6.22 (3.18), 5 [4–7]	5.87 (2.50), 5 [4–7]
<5	165 (35.64%)	175 (37.00%)
≥5	298 (64.36%)	298 (63.00%)
Systolic blood pressure, mmHg	146.34 (22.99)	146.21 (20.87)
	142 [130–160]	142 [130–159]
Diastolic blood pressure, mmHg	85.30 (13.57)	85.28 (11.99)
	82 [77–92]	83 [79–91]
Blood glucose, mmol/L	7.06 (3.37)	7.01 (3.38)
	5.83 [5.08–7.88]	5.75 [5.08–7.74]
Medical history		
Hypertension	395 (83.86%)	397 (83.05%)
Diabetes	196 (41.61%)	190 (39.75%)
Dyslipidemia	23 (4.88%)	22 (4.60%)
Atrial fibrillation	4 (0.85%)	3 (0.63%)
Severity of ICAS Stenosis^a^		
<30%	94 (31.02%)	83 (26.69%)
30%–50%	24 (7.92%)	16 (5.14%)
51%–70%	64 (21.12%)	81 (26.05%)
71%–99%	121 (39.93%)	131 (42.12%)
Site of ICAS^b^		
ACA	25 (11.96%)	32 (14.04%)
MCA	129 (42.57%)	151 (66.22%)
PCA	65 (31.10%)	58 (25.44%)
ICA	92 (44.02%)	120 (52.62%)
VA/BA	40 (19.14%)	52 (22.81%)

Note

Data are presented by n (%), mean (SD) or median (IQR).

Abbreviations: ACA, anterior cerebral artery; ICA, intracranial carotid artery; ICAS, intracranial arterial stenosis; MCA, middle cerebral artery; NIHSS, National Institution of Health Stroke Scale;PCA, posterior cerebral artery; VA/BA, vertebral artery/basilar artery.

^a^ Only 303 patients in GISAA group and 311 patients in control groups had the central imaging assessment.

^b^ Only patients with largest stenosis severity >30% were counted for the site of ICAS, therefore, only 209 patients in GISSA group and 228 patients in control group.

The primary outcome was observed in none of the 463 patients in the ginkgolide group, but in 6 of 473 patients in the placebo group. These patients had recurrent stroke within 28 days of onset. None died in either of the groups. Ginkgolide was associated with less recurrent stroke in ICAS patients (0/463 vs. 6/473, risk ratio of non‐event index 1.013, 95% CI 1.003–1.023, *p *= 0.031).

**FIGURE 2:**
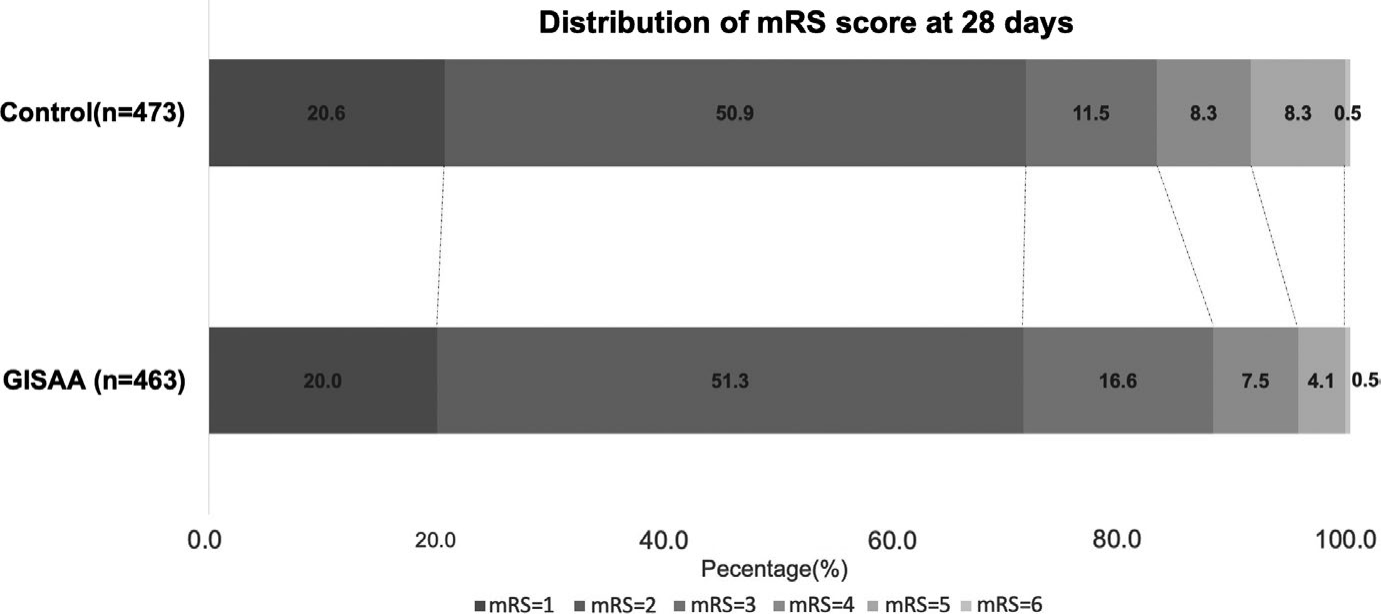
Function outcome measured by modified Rankin Scales of all patients

We also obtained both NIHSS and mRS scores in 414 (89.4%) patients in the ginkgolide group and in 433 (91.5%) patients in the placebo group at day 28 follow‐up. Favorable outcome (mRS ≤ 2) was observed in 362/463 (78.2%) patients in the ginkgolide group and 362/473 (76.5%) patients in the placebo group, as intention‐to‐treat analysis (Table 2). There was an association between the use of ginkgolide and favorable outcome (RR 1.492, 95% CI 1.013–2.198). Similar improvement trend was observed on neurological deficits measured by NIHSS (Table 2). The distribution of mRS showed that the patients in the GISAA group were more likely to have a better functional outcome compared with those in the control group (Figure 2).

**TABLE 2:** Comparison of primary outcome and secondary outcome

Variable	Ginkgolide *N* = 463	Placebo *N* = 473	RR	95% CI	*p* value
Primary outcome^a^	0 (0.00%)	6 (1.27%)	1.013	1.002–1.023	0.031
Mortality	0 (0.00%)	2 (0.42%)	1.004	0.998–1.010	0.500
Recurrent stroke	0 (0.00%)	4 (0.85%)	1.008	1.002–1.017	0.124
SAE	5 (1.08%)	3 (0.63%)	1.703	0.409–7.084	0.502
Secondary outcome at 28 days					
mRS 0–1	293/463 (71.3%)	312/473 (71.5%)	0.989	0.734–1.331	0.940
mRS 0–2	362/463 (78.2%)	362/473 (76.5%)	1.492	1.013–2.198	0.042
NIHSS improvement^b^	3.73 ± 2.24	3.36 ± 2.28	0.370	0.071–0.681	0.016
Platelet aggregation pathways results at 14 days					
PAF, pg/ml^b^	285.72 ± 276.05	347.75 ± 489.79	−62.030	−120.593– −3.467	0.036
ADP, ng/ml^b^	294.21 ± 254.52	319.32 ± 387.75	−25.104	−73.351–23.142	0.304
TAX2, pg/ml^b^	1314.02 ± 2893.23	1244.34 ± 2629.64	70.012	−334.775–474.499	0.734

Note

Abbreviations: ADP, adenosine‐5’‐diphosphate (ADP); CI, confidential interval; mRS, modified Rankin Scale; NIHSS, National Institution of Health Stroke Scale; PAF, platelet aggregation factor; RR, risk ratio; SAE, severe adverse event; TXA2, thromboxane A2.

^a^ Composite index event was consisted with recurrent ischemic stroke, hemorrhage stroke, and mortality caused by vascular event.

^b^ The NIHSS improvement between the baseline NIHSS and the performance at 28 days and all levels of platelet aggregation pathway at 14 days were presented by 95% CI difference and analyzed by linear regression, presented with mean ± SD and coef.

**FIGURE 3:**
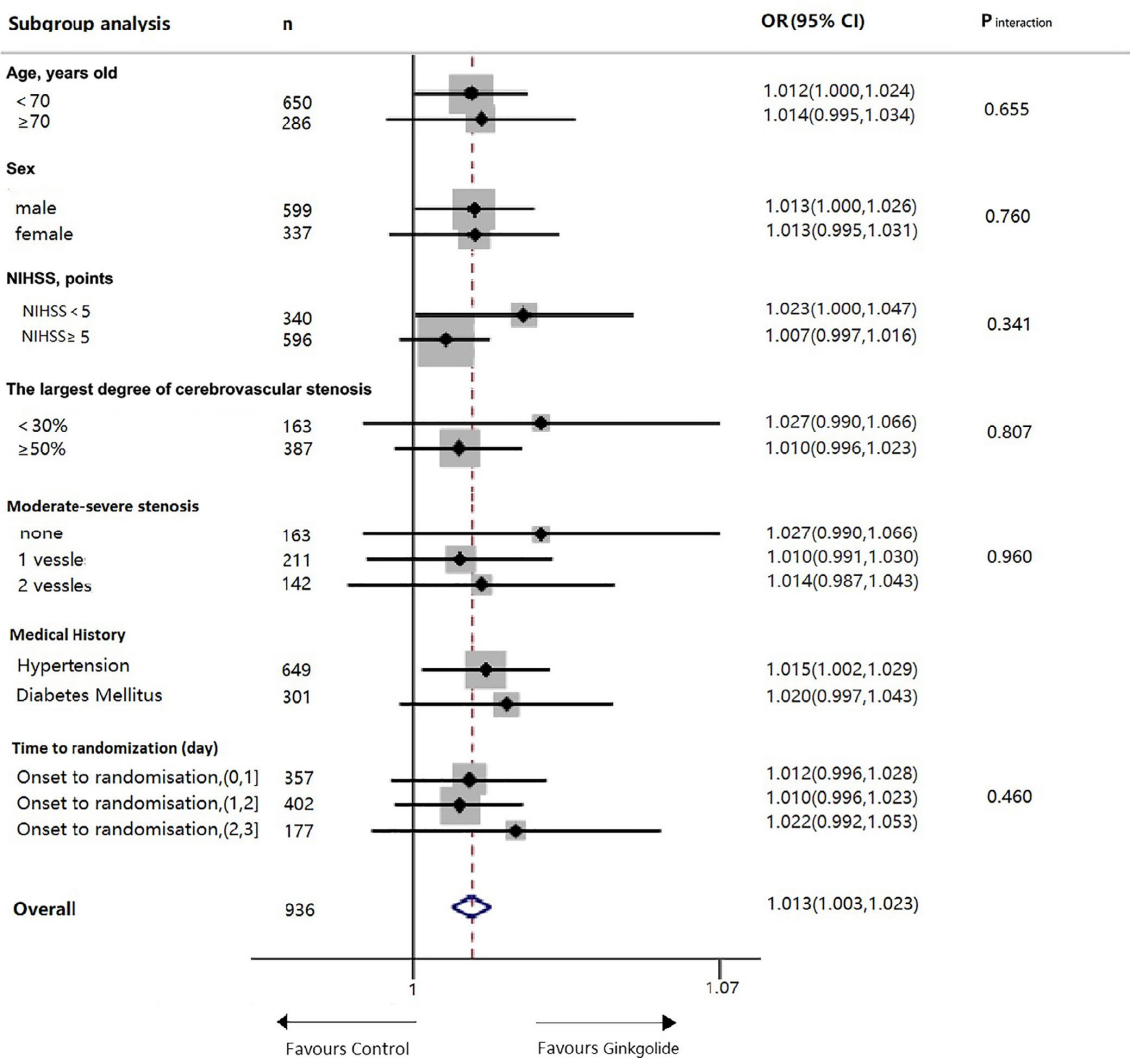
Specified Subgroup analysis of the primary outcome. NIHSS, National Institution of Health Stroke Scale. Center‐imaging assessment was performed among 299 in Ginkgolide group and 307 in placebo group who has DICOM information. According to NACET criteria, CTA or MRA imaging was classified into mild (0%–30%), moderate (30%–50%), and severe stenosis (>50%). The patients with 30%–50% stenosis had no events. Therefore, there is no RR for these specific patients

The specified subgroups analysis of index events was conducted and is demonstrated in Figure 3. There was no significant difference in specified subgroups.

AE was observed in 159/463 in the ginkgolide group and in 171/473 in the placebo group. SAE occurred in 5/463 patients in the ginkgolide group and in 3/473 patients in the placebo group. There was no significant association between the use of ginkgolide and SAE. Of note, there was one intracranial hemorrhage event in the placebo group.

**FIGURE 4:**
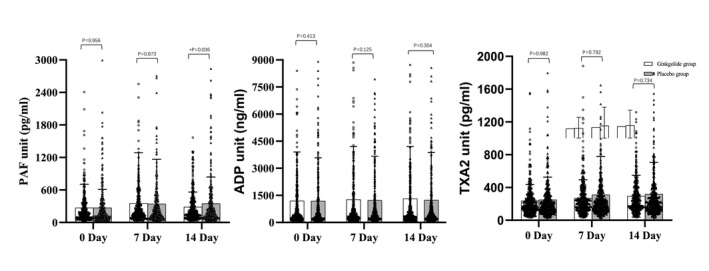
Comparison of platelet reactivities in different activating pathways at baseline and follow‐up visits. ADP, adenosine‐5’‐diphosphate (ADP); PAF, platelet aggregation factor; TXA2, thromboxane A2. Platelet reactivities in different pathways in trial groups at baseline and follow‐up, showing PAF was inhibited by Ginkgolide, while other traditional TAX2 and ADP levels were not associated with Ginkgolide treatment

Platelet aggregation functions, including PAF, TXA2, and ADP, were measured in both ginkgolide and placebo groups. The data concerning the level of PAF, TXA2, and ADP were tested for normality (Table S1). A total of 370, 367, and 352 patient samples in the ginkgolide group and 386, 380, and 366 patient samples in the placebo group were included in day 0, 7, and 14 analyses, respectively. Using linear regression model, we observed that there was a reduction in the PAF level in the ginkgolide group after adjustment of age, onset of time, sex, and the severity at baseline (*p *= 0.036, adjusted *p *= 0.035). There were no similar trends found in TXA2 and ADP pathway (Figure 4).

## 4 DISCUSSION

Our study showed that ginkgolide had a slight benefit in moderate‐to‐severe AIS patients with ICAS. Furthermore, ginkgolide was also associated with functional outcome (mRS 0–2) improvement at day 28 follow‐up compared with those in the placebo group. By assessing the various platelet‐activating pathways, the effect of intravenous ginkgolide was associated with PAF inhibition in AIS patients.

Ginkgolide used in this study, playing a role as an inhabitation of PAF receptor, was induced by ischemic stroke.^10^ The reduction in PAF and its pathway were reportedly helpful to reduce the volume of infarction in acute phase.^9^, ^10^, ^11^ In our study, the dynamic changes in PAF of both groups were confirmed, which is consistent with the recurrent stroke risk. Ginkgolide helped slightly in decreasing accumulation of PAF after ischemic stroke, which might be one of the mechanisms.

AIS patients with ICAS had a higher risk of recurrent stroke in acute phase,^3^, ^14^ which was challenging^15^]. Based on previous studies, ICAS is still one of the most challenging conditions in AIS patients. The standard treatment of ICAS, especially in acute phase, is debated.^16^ Based on previous CHANCE trial, aspirin plus clopidogrel in mild stroke with ICAS is recommended.^5^ However, this combination was not superior to aspirin monotherapy in patients with ICAS in the CHANCE substudy.^17^ The POINT trial showed that the dual antiplatelet treatment might cause more bleeding events^7^]. Moreover, only mild stroke patients were included in the above studies. In our study, we focused on those patients with moderate stroke, which might be more complicated.

On the one hand, other antithrombotic treatments were also explored.^16^ Combining the Trial of Cilostazol in Symptomatic Intracranial Arterial Stenosis (TOSS) study and TOSS II trial, aspirin plus cilostazol in ICAS patients showed better clinical outcomes with the overall change in stenosis compared with aspirin only.^18^, ^19^ On the other hand, the Vitesse Stent Ischemic Therapy Trial showed that the use of a balloon‐expandable stent compared with medical therapy resulted in an increased 12‐month risk of added stroke or TIA in the same territory, and increased the 30‐day risk of any stroke or TIA.^20^ Similarly, SAMMPRIS trial showed that the early benefit of aggressive medical management over stenting with the Wingspan stent for high‐risk patients with intracranial stenosis persisted over extended follow‐up.^1^ However, The Wingspan Stent System Post Market Surveillance and other post‐marketing studies showed a great improvement in the intervention group recently.^21^, ^22^ Our study focused on the same symptomatic ICAS patients and found that ginkgolide was of benefit in acute phase stroke patients and without bleeding risk, compared with that of aspirin along. Compared with other combinations of antiplatelet agents, ginkgolide, as a PAF inhibitor, had an advantage of less bleeding risk.^23^, ^24^ Our study also confirmed that the effect of ginkgolide was not associated with ADP and TXA2 pathway.

Our study had several limitations. First, the diagnosis of AIS patients with ICAS was based on the on‐site treating physician's judgment. Due to the short enrollment time window, we could not perform the assessment by the radiologists at the imaging center before randomization. Consequently, about one‐fourth of patients enrolled had actually mild stenosis when their images were interpreted at the central imaging center. Therefore, only central imaging results were used in final analysis. Second, unexpectedly low rate of index events might have affected the exploration of benefit compared with the CHANCE trial subgroup analysis.^5^ The “ceiling effect” might be influenced on the effect of primary outcome, while there was no effect on the secondary functional outcome. Third, many other risk factors, as confounders, might be influenced on the recurrence in ICAS patients, such as the residual flow, leptomeningeal collateral flow, and hemispheric blood flow, which were supposed to be balanced at randomization; we failed to analyze these factors in our study.^25^, ^26^ Finally, the underlying mechanisms and their pathways of other components within ginkgolide need further studies on mechanism. Although our study showed a slight benefit in the composite index event prevention for the primary outcome, a larger scale with longer follow‐up duration RCT trial is needed to confirm this finding.

In this trial, intravenous ginkgolide was safe and had a benefit in recurrent stroke prevention in AIS patients with ICAS. It might also be associated with functional improvement in a short‐term follow‐up, which could be considered as an optional choice in disabling stroke patients with ICAS at acute phase.

## CONFLICT OF INTERESTS

All authors report no disclosures. All authors have completed and submitted the ICMJE Form for Disclosure of Potential Conflicts of Interest.

## AUTHOR CONTRIBUTIONS

Qiang Dong, Huashan Hospital, Fudan University, Primary Investigator, Concept of the study and framed the study; Revised the manuscript. Yi Dong, Huashan Hospital, Fudan University, Data analysis, drafted the manuscript, Performed biostatistical analysis and drafted the manuscript. Jingyu Zhang, The Fourth Affiliated Hospital, Harbin Medical University; Yanxia Wang, People's Hospital, Hejian; Lihong Zhao, People's Hospital, Dandong; Runhui Li, Affiliated Central Hospital of Shenyang Medical College. Shenyang; Chunhua Wei, Affiliated Nanshi Hospital, Henan University; Qingke Bai, Pudong New Area People's Hospital, Shanghai; Lishu Wan, Dandong First Hospital, Dandong; Liping Sun, Pingdingshan Municipal Second People's Hospital, Pingdingshan; Shejun Feng, Handan Central Hospital, Hebei; Mingyao You, Guizhou Medical University affiliating Hospital; Chun Wang, The First People's Hospital, Ruzhou; Hongtian Zhang, People's Hospital, Zhecheng; Qing He, Transportation Center Hospital, Yunnan;. Ming Yu, Jiangsu University affiliating Hospital, Site Investigator, Reviewed the manuscript and coordinated the study on site.

## EXCLUSIVE LICENSE

The corresponding author has the right to grant on behalf of all authors.

## PATIENT AND PUBLIC INVOLVEMENT

Partly patient and public were involved in our study. We will write a plain language summary and design a leaflet for dissemination to their peers and distribute to patient groups after publishing.

## Supporting information

Table S1

## Data Availability

The technical appendix, dataset, and statistical code are available from the corresponding author at dong_qiang@fudan.edu.cn. The lead author affirms that this manuscript is an honest, accurate, and transparent account of the study being reported; that no important aspects of the study have been omitted; and that any discrepancies from the study as planned (and, if relevant, registered) have been explained.

## APPENDIX 1

### Co‐investigators

Lei Huang, Huashan Hospital, Fudan University; Wenjie Cao, Huashan Hospital, Fudan University, Review imaging for study, independently reviewed the imaging. Dongjuan Xu, Dongyang People's Hospital, Zhejiang Province; Jianjun Guo, Zengcheng People's Hospital, Guangzhou; Jiadong Zhang, Harbin The First Hospital, Harbin; Guofang Chen, Xuzhou Central Hospital; Yan Wei, Harrison International Peace Hospital (Hengshui City People's Hospital); Shuangxing Hou, Pudong Hospital, Fudan University; Xijing Mao, The Second Hospital, Jilin University; Rongxia Ji, Henan General Hospital; Jifa Long, Nanchong Central Hospital, Nanchong; Caixiao Chen, Traditional Chinese Medicine Hospital, Xinle; Yong Zhao, Traditional Chinese Medicine Hospital, Haicheng; Guozhong Li, The First Affiliated Hospital, Harbin Medical University; Wenjun Yu, Yudong Hospital of the First Affiliated Hospital of Henan University of TCM; Jianhua Xu, Jiading District Central Hospital Affiliated Shanghai University of Medicine & Health; Xiangyu Pu, Affiliated Zhongshan Hospital, Dalian University; Xiaohong Li, Jinan Central Hospital; Zhilin Jiang, 202nd Hospital of People's Liberation Army, Shenyang; Yi Yang, The First Hospital, Jilin University; Wei Li, Affiliated Hospital, Beihua University; Qingyou Zeng, The Second People's Hospital, Huludao; Tao Sun, Traditional Chinese Medicine Hospital, Qiuxian; Yingmin Song, Baoshen Wang, Traditional Chinese Medicine Hospital, Luohe; Guiru Zhang, People's Hospital, Penglai; Huisheng Chen, Shenyang Military Region General Hospital, Shenyang; Junyan Liu, The Third Hospital, Hebei Medical University; Zhenguo Liu, Xinhua Hospital Affiliated to ShangHai JiaoTong University School of Medicine; Yongge Hou, The First Hospital, Shijiazhuang; Biyun Zong, Heze Municipal Hospital; Jun Liu, The First Hospital, Kunming; Bihua Wu, Affiliated Hospital of North Sichuan Medical College; Xin Wang, Zhongshan Hospital, FuDan University; Ding Qin, Jingmei Group General Hospital, Beijing; Ming Zhang, Traditional Chinese Medicine Hospital, Jingmen; Weizhong Gu, The Second People's Hospital, Datong; Houqin Chen, The First People's Hospital, Wuhu; Qing He, Xuzhou No.1 People's Hospital; Anding Xu, The First Affiliated Hospital of Medical College, Jinan University; Yun Xu, Nanjing Drum Tower Hospital, The Affiliated Hospital of Nanjing University Medical School; Mingzong Yan, Traditional Chinese Medicine Hospital, Penglai; Xiaoya Feng, Provincial Third Hospital, Shandong; Jun Tan, The Third Affiliated Hospital, Xinxiang Medical University; Ping Sun, The Second People's Hospital, Guiyang; Shaohua Su, People's Hospital, Dezhou; Zhengqi Lu, The Third Affiliated Hospital, Sun Yat‐sen University; Xiao Bo, Xiangya Hospital, Central South University; Zhen Jiao, Anshan Central Hospital, Site Investigator, coordinated the study on site.
