# Supplementary material for: Effect of Ginkgolide in Ischemic Stroke patients with large Artery Atherosclerosis: Results from a randomized trial
Source: CNS Neurosci Ther. 2021 Oct 22;27(12):1561–9. doi: 10.1111/cns.13742 (PMC8611772; doi:10.1111/cns.13742)
Supplement: Supplementary file 1 — Table S1 [file CNS-27-1561-s001.docx]

**Supplemental Table 1**

**The tests for normality among platelet-aggregation pathways**

|  | Group | Tests for Normality | Wilcoxon Test Between Two Groups |
| --- | --- | --- | --- |
| **Baseline** |  |  |  |
| PAF | Ginkgolide group | W=0.413227 P<0.0001 | Z=0.2235  P=0.8231 |
|  | Placebo group | W=0.640738 P<0.0001 |  |
| TXA2 | Ginkgolide group | W= 0.378522 P<0.0001 | Z= -0.5862  P= 0.5577 |
|  | Placebo group | W= 0.432823 P<0.0001 |  |
| ADP | Ginkgolide group | W=0.737053 P<0.0001 | Z=0.2126  P=0.8317 |
|  | Placebo group | W=0.570306 P<0.0001 |  |
| **Day 7** |  |  |  |
| PAF | Ginkgolide group | W=0.237798 P<0.0001 | Z=-0.2317  P=0.8168 |
|  | Placebo group | W=0.253789 P<0.0001 |  |
| TXA2 | Ginkgolide group | W= 0.373498 P<0.0001 | Z= -0.9575  P= 0.3383 |
|  | Placebo group | W= 0.413099 P<0.0001 |  |
| ADP | Ginkgolide group | W=0.737749 P<0.0001 | Z=0.4743  P=0.6353 |
|  | Placebo group | W=0.407877 P<0.0001 |  |
| **Day 14** |  |  |  |
| PAF | Ginkgolide group | W=0.772317 P<0.0001 | Z=-0.3434  P=0.7313 |
|  | Placebo group | W=0.56645 P<0.0001 |  |
| TXA2 | Ginkgolide group | W= 0.392132 P<0.0001 | Z= -0.1478  P= 0.8825 |
|  | Placebo group | W= 0.408552 P<0.0001 |  |
| ADP | Ginkgolide group | W= 0.719269 P<0.0001 | Z= 0.6790  P= 0.4971 |
|  | Placebo group | W= 0.536292 P<0.0001 |  |

PAF=platelet aggregation factor. TXA2=thromboxane A2. ADP=adenosine-5’-diphosphate (ADP)
